# Differing Requirements for RAD51 and DMC1 in Meiotic Pairing of Centromeres and Chromosome Arms in *Arabidopsis thaliana*

**DOI:** 10.1371/journal.pgen.1002636

**Published:** 2012-04-19

**Authors:** Olivier Da Ines, Kiyomi Abe, Chantal Goubely, Maria Eugenia Gallego, Charles I. White

**Affiliations:** Génétique, Reproduction et Développement, UMR CNRS 6293, Clermont Université, INSERM U1103, Aubière, France; Cornell University, United States of America

## Abstract

During meiosis homologous chromosomes pair, recombine, and synapse, thus ensuring accurate chromosome segregation and the halving of ploidy necessary for gametogenesis. The processes permitting a chromosome to pair only with its homologue are not fully understood, but successful pairing of homologous chromosomes is tightly linked to recombination. In *Arabidopsis thaliana*, meiotic prophase of *rad51*, *xrcc3*, and *rad51C* mutants appears normal up to the zygotene/pachytene stage, after which the genome fragments, leading to sterility. To better understand the relationship between recombination and chromosome pairing, we have analysed meiotic chromosome pairing in these and in *dmc1* mutant lines. Our data show a differing requirement for these proteins in pairing of centromeric regions and chromosome arms. No homologous pairing of mid-arm or distal regions was observed in *rad51*, *xrcc3*, and *rad51C* mutants. However, homologous centromeres do pair in these mutants and we show that this does depend upon recombination, principally on DMC1. This centromere pairing extends well beyond the heterochromatic centromere region and, surprisingly, does not require XRCC3 and RAD51C. In addition to clarifying and bringing the roles of centromeres in meiotic synapsis to the fore, this analysis thus separates the roles in meiotic synapsis of DMC1 and RAD51 and the meiotic RAD51 paralogs, XRCC3 and RAD51C, with respect to different chromosome domains.

## Author Summary

Meiosis is a specialised cell division that acts to halve the chromosome complement, or ploidy, in the production of gametes for sexual reproduction in eukaryotes. To ensure that each gamete has a full complement of the genetic material, homologous chromosomes must pair and then separate in a coordinated manner during meiosis, and this is mediated by recombination in the majority of studied eukaryotes. To better understand the relationship between recombination and meiotic homologue pairing, we have analysed meiotic chromosome pairing in plant mutants lacking key recombination proteins. This work provides new insights into the homologous chromosome pairing mechanisms occurring in meiotic prophase of *Arabidopsis thaliana*: heterochromatic centromeres and 5S rDNA regions pair early, and their pairing has different requirements for recombination proteins than does that of the chromosome arms. These data raise a number of questions concerning the specificities and roles of recombination at different chromosome and/or chromatin regions in the synapsis of homologous chromosomes at meiosis.

## Introduction

Sexual reproduction involves the fusion of maternal and paternal gametes and this means that the parental genetic complement must be halved in the process of gametogenesis, to avoid it doubling at each generation. This halving of ploidy is carried out by meiosis, the specialised eukaryotic cell division that involves one round of DNA replication followed by two sequential divisions. Errors in segregation of the genetic material during cell division lead to aneuploidy, a well known example of which in humans is Down's Syndrome, caused by trisomy of chromosome 21 [1]. It is thus essential that each daughter cell inherits a full complement of the genetic material and in mitosis this is ensured by centromeric cohesion established at the preceeding S-phase. This mechanism also ensures proper chromosomal segregation during the second meiotic division, however recognition and linking of homologous chromosomes at the first meiotic division is mediated in the majority of eukaryotes by recombination during the first meiotic prophase.

While understanding of the mechanisms of homologous recombination has considerably advanced, the processes permitting chromosomes to recognise and pair with only their homologues remain elusive. Homologue pairing has been shown to depend on a number of mechanisms including homologous recombination, centromere coupling, telomere clustering and the interaction of specific pairing centres with Zinc finger DNA-binding proteins seen in *Drosophila melanogaster* and *Caenorhabditis elegans* [2]–[6]. In animals and fungi, clustering of telomeres at the leptotene/zygotene transition into a “bouquet” associated with the microtubule organising center promotes homologue alignment (reviewed by [7]). Evidence from budding yeast shows that Zip1- and Rec8-dependent centromere coupling, or non-homologous centromere pairing, precedes homologous interactions, which are then stabilised by Spo11-dependent homologous recombination mechanisms [8]–[11]. A recent report has shown that PP4 phosphatase is required to counteract Mec1-dependent phosphorylation of Zip1 and permit this non-homologous centromere coupling [12]. Transition to homologous chromosome interactions leads to co-alignment of homologous chromosome axes in zygotene, and the fully synapsed chomosomes visible at pachytene. In wheat, centromeres cluster premeiotically and further associate in pairs facilitating pairing and recombination in a mechanism dependent on the Ph1 locus [13], [14] and centromere coupling is observed in the *Arabidopsis thaliana phs1* mutant [15]. The role of centromeres in meiotic pairing is an active subject of research in many organisms (reviewed by [16]). In this context it is important to note that the structure of centromeric regions differs considerably between species, ranging from 125 bp in *Saccharomyces cerevisiae* to the highly repeated DNA of up to several megabases in length found in multicellular eukaryotes. In Arabidopsis, centromeric DNA contains long stretches of tandemly repeated DNA sequences, transposons, retrotransposons and rDNA, and this has meant that full DNA sequence analysis of Arabidopsis centromeres has not been completed [17]–[20]; see review by [21].

Meiotic recombination is initiated by the induction of double-strand DNA breaks in the chromosomes by Spo11 during the leptotene stage. Resection at the DSB generates single-stranded DNA overhangs and these load Rad51 and/or Dmc1 protein into a helical nucleofilament, which catalyses invasion of, and synapsis with, an homologous DNA sequence. Repair of DSB in G2 and M-phase mitotic cells, and the majority of DSB in meiotic cells, involves primarily the invasion of the sister chromatid. However during meiosis, a subset of breaks are repaired through recombination with the homologous chromosome, thus establishing the physical linkage necessary to ensure proper chromosomal disjunction at the first meiotic anaphase. How meiotic cells permit synapsis with sister chromosome, rather than the sister chromatid, is a major question in meiosis and much research is currently focussed on the specificities of the Rad51 recombinase and its meiosis-specific paralogue, Dmc1. Dmc1 has been shown to play similar, but not identical roles to Rad51 [22], however, while Rad51 is needed for both meiotic and mitotic recombination, Dmc1 is only required in meiosis [5], [23]. In yeast, current understanding points to action of the Red1/Hop1/Mek1 complex promoting meiotic inter-homolog recombination through phosphorylation of the axial element protein Hop1 [24]–[26]. Also, Hed1 restricts activity of Rad51 nucleofilaments in meiosis by blocking access of Rad54 [27], [28]. These mechanisms attenuate the activity of Rad51 to minimise the use of the sister chromatid and hence favour Dmc1-dependent inter-homolog recombination. Dmc1 plays a key role in inter-homolog recombination in plants, yet the mechanisms through which RAD51 and DMC1 cooperate to promote the homology search and chromosome synapsis are unknown [29]–[31]. Arabidopsis mutants lacking RAD51 or DMC1 are sterile but show normal somatic growth [29], [32]. Both mutants show defects in pairing and synapsis, however while *rad51* mutants exhibit extensive chromosome fragmentation at late pachytene, *dmc1* mutants are characterised by the presence of intact univalents, showing that DSBs are repaired in a RAD51-dependent manner using the sister chromatid as a template in the absence of DMC1 [33]. The precise roles and mechanisms of action of Dmc1 nonetheless remain poorly understood and some organisms, such as Drosophila or *Caenorhabditis elegans*, do not possess an apparent Dmc1 ortholog.

The RAD51 paralogue proteins RAD51C and XRCC3 play key roles in the homology search together with RAD51, and have been shown to act as cofactors facilitating loading of RAD51 onto DNA [34]–[39]. The lethality of these mutants in vertebrates has hampered the study in meiosis and the details of their roles in meiotic homologous pairing remain elusive [40]–[42]. In Arabidopsis, *xrcc3* and *rad51C* mutants are viable but extensive chromosome fragmentation in late pachytene results in sterility, a similar phenotype to that of *rad51* mutants [43]–[47]. Both the RAD51C and XRCC3 proteins are essential for meiotic recombination and they have both early and late functions during meiotic prophase I [40]–[49]. The specific role(s) of XRCC3 and RAD51C in homologous recombination and how they cooperate with the RAD51 and DMC1 recombinases to achieve efficient homologous chromosome pairing remain subject to debate.

Working with the flowering plant *Arabidopsis thaliana*, we present here an analysis of the roles of recombination and key recombination proteins in this process, showing the existence of a DMC1-dependent process which stabilises pairing of centromeric regions of homologous chromosomes and that, in contrast, synapsis of chromosome arms requires RAD51 protein and the RAD51 paralogues, RAD51C and XRCC3.

## Results

### Arabidopsis *xrcc3* and *rad51C* Recombination-Defective Mutants Exhibit Partial Chromosome Alignment and Pairing

Meiosis in Arabidopsis *xrcc3* and *rad51C* mutants progresses up to the zygotene stage and appears to enter pachytene, but do not show full chromosomal synapsis at pachytene and we thus call this meiotic stage zygo-pachytene. Subsequent meiotic stages present fragmented and fused chromosomes and no viable gametes are produced [43]–47,50. The presence of pachytene-like figures and the observation of fragmented bivalents at metaphase I show that some chromosome pairing does occur in these mutants and we have set out to determine the nature of this and its dependence on recombination.

**Figure 1:**
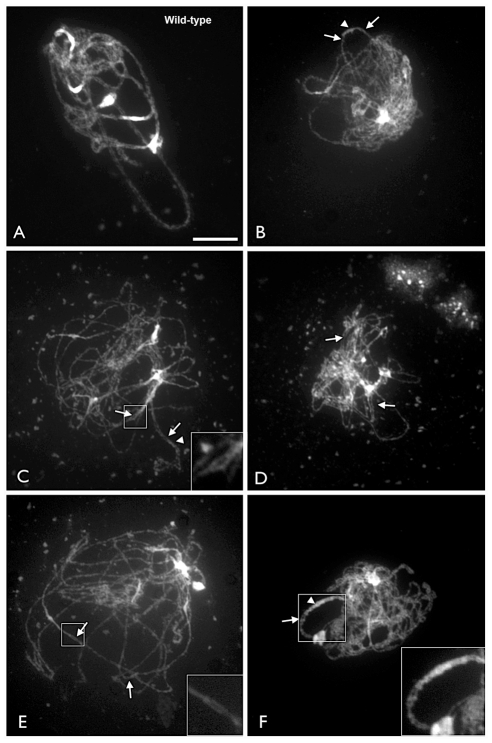
Arabidopsis *xrcc3* mutants exhibit partial chromosome alignment and pairing. Chromosome spreads of zygo-pachytene of WT (A) and *xrcc3* mutants (B to F) obtained from chromosome spreads counterstained with DAPI. Arrowheads point to paired/aligned chromosomes and thick fibres characteristic of normal synapsed chromosomes, while arrows depict loop structures corresponding to unpaired regions. Insets in C, E, and F are magnifications of the boxed areas (white square) showing loop structures. (Scale bar: 10 µm.)

Observation of meiotic figures clearly confirms the presence of partial synapsis at the zygo-pachytene stage in *xrcc3* mutants. Chromosome alignment and pairing is visible as short stretches and thick chromosome threads typical of synapsed chromosomes are clearly visible (Figure 1). The thick, synapsed fibres frequently end in bubble structures in which the two chromosome axes are clearly visible (Figure 1B–1F). Immunolocalisation of the synaptonemal complex (SC) central element protein, ZYP1, shows foci and short stretches in the *xrcc3* mutant, in agreement with the observed partial synapsis of DAPI-stained chromosomes (Figure S1). Similar ZYP1 staining patterns have been described in Arabidopsis *rad51*, *xrcc3*, and *rad51C* mutants [50]. Transmission electron microscopy studies in *rad51* and *rad51C* mutants also show short sections of synaptonemal complex and thus evidence of partial chromosome synapsis [32], [47].

### Pairing of Chromosome Arms Is Dependent on Xrcc3 and Rad51C

**Figure 2:**
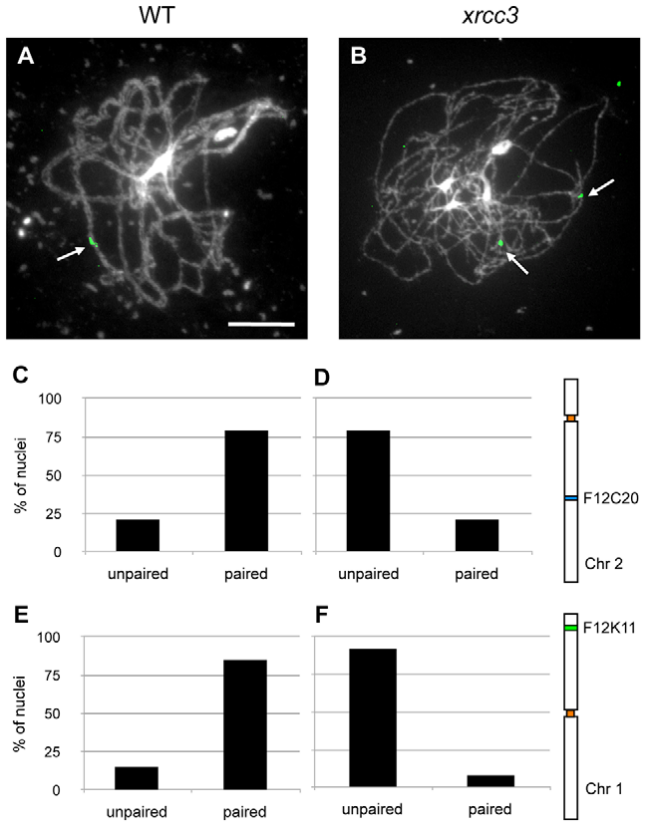
Chromosome arms do not synapse in the *xrcc3* mutant. FISH using BAC probe F12C20 targeting an euchromatic region of the long arm of chromosome 2 (green). Images of WT pachytene showing a single FISH signal (arrowed) representative of paired chromosomes (A) and *xrcc3* zygo-pachytene showing two FISH signals (arrowed) (B). DNA is stained with DAPI. (Scale bar = 10 µm.). C to F. Percentages of WT (C,E) pachytene and *xrcc3* (D,F) zygo-pachytene nuclei showing one (paired) or two (unpaired) FISH signals. Probe in C,D is interstitial BAC F12C20 on chromosome 2. Probe in E,F is distal BAC F12K11 on chromosome 1 (see schemas to right).

These observations prompted us to further characterise the partial synapsis observed in the *rad51* paralogue mutants, using fluorescence *in situ* hybridisation (BAC-FISH) to examine pairing in *xrcc3* mutants using a BAC probe (F12C20) recognising a mid-arm region of the long arm of chromosome 2 (Figure 2). As expected, in wild-type meiocytes, two separate signals were observed from leptotene to early zygotene and one signal (or two paired signals) were observed in pachytene figures (Figure 2 and Table 1). In contrast, in *xrcc3* mutants two signals were observed in 80 percent of the meiocytes at the zygo-pachytene stage (Figure 2 and Table 1). Similar results were obtained using a second BAC probe (F12K11) probing the distal region of the left-arm (2 Mbp from the telomere) of chromosome 1, with more than 90% of *xrcc3* meiocytes showing unpaired chromosomes while 85% of the WT meiocytes were fully paired (Figure 2 and Table 1). BAC-FISH analyses in *rad51C* meiocytes using F12K11 probe confirmed the absence of pairing observed in the *xrcc3* mutant (Table 1). Pairing and synapsis in these regions is thus dependent on RAD51 paralogue-dependent recombination.

**Table 1:** Chromosome arm pairing during meiotic prophase I in WT, *xrcc3*, and *rad51C* plants.

	Euchromatic probe F12C20				Euchromatic probe F12K11			
	Leptotene		Zygo-pachytene		Leptotene		Zygo-pachytene	
	unpaired	paired	unpaired	paired	unpaired	paired	unpaired	paired
WT	95% (18)	5% (1)	21% (3)	79% (11)	100% (11)	0% (0)	15% (10)	85% (56)
*xrcc3*	97% (35)	3% (1)	79% (23)	21% (6)	90% (9)	10% (1)	92% (150)	8% (13)
*rad51C*					100% (7)	0% (0)	97% (29)	3% (1)

Percentages of WT, *xrcc3*, or *rad51C* meiocytes showing paired or unpaired signals in FISH using mid-arm probes on chromosomes 2 or 1. Numbers of nuclei are given in brackets.

### Centromeres Pair in Arabidopsis *xrcc3* and *rad51C* Mutants

**Figure 3:**
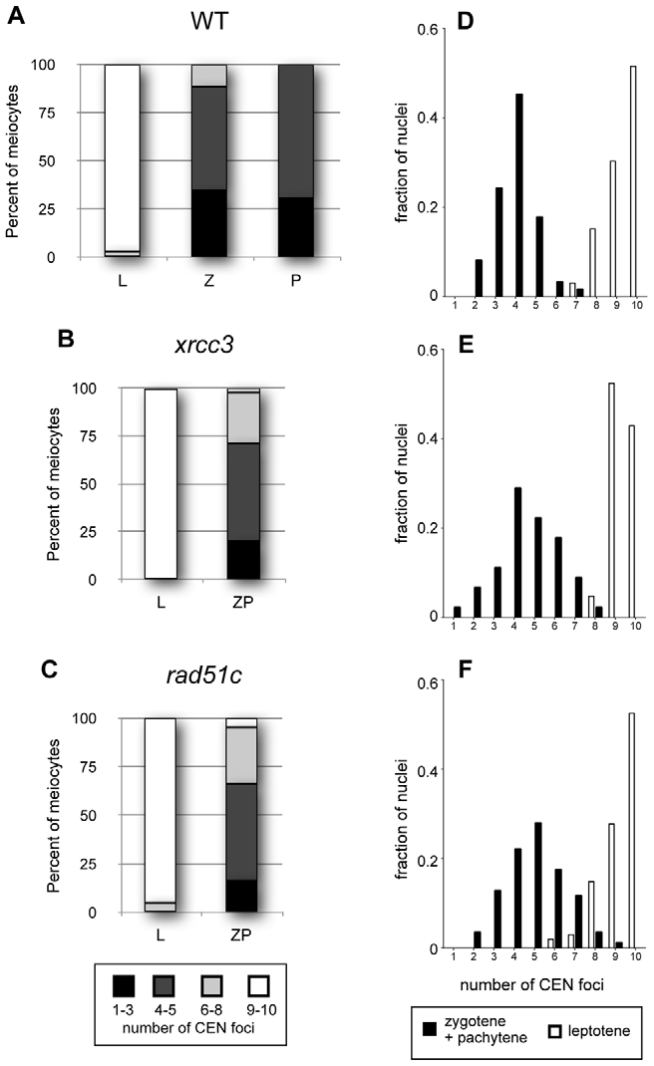
Centromere pairing in Arabidopsis *xrcc3* and *rad51C* mutants. Distributions of centromeric FISH signals in prophase I nuclei of wild-type (A and D, n = 95), *xrcc3* (B and E, n = 66) and *rad51C* (C and F, n = 273) mutant plants. Since full pachytene figures are not observed in the mutants, zygotene and pachytene-like figures were grouped in the zygo-pachytene class. (A to C) : progression in centromere coupling through prophase I with 8 to 10 (fully unpaired), 1 to 3 (clustering), 6 to 7 (partially paired) and 4 or 5 centromeres (complete coupling). (D to F) Frequency distributions of centromeric signals in WT (D), *xrcc3* (E) and *rad51C* (F).

Centromere pairing is an early event in meiotic chromosome pairing and has been well described in Arabidopsis [51]. Arabidopsis centromeres are unpaired and dispersed during meiotic interphase up to leptotene, cluster at leptotene/zygotene, separate and homologous centromeres then associate in pairs and synapse in zygotene and pachytene. FISH analyses using a 180 bp centromeric repeat-specific probe were used to follow pairing of centromeres through meiotic prophase I in *xrcc3* and *rad51C* mutants (Figure 3). For each stage, meiocytes were placed into one of four classes according to the number of fluorescent signals from the centromeric repeat-specific probe : in type 1, 8–10 centromere signals indicate unpaired centromeres; in type 2 meiocytes, 6–7 signals show partial centromere pairing; type 3 meiocytes have clustered centromeres (1 to 3 signals) and type 4 meiocytes show four or five signals and complete centromere pairing (Figure 3).

In wild- type, virtually all leptotene nuclei were of type 1 (unpaired centromeres; Figure 3A and 3D). Zygotene nuclei were mostly of type 3 or 4, while in pachytene 69% of nuclei were of type 4 and the remainder of type 3, indicative of complete centromere pairing (Figure 3A and 3D). Similarly, in *xrcc3* and *rad51C* mutants, more than 95 percent of the observed leptotene nuclei showed ten centromere signals (type 1 nuclei). The proportion of Type 1 nuclei then declined to reach the five expected signals (type 4) in zygo-pachytene, *xrcc3* and *rad51C* show centromere pairing with 51% and 50% type 4 nuclei, respectively (Figure 3B, 3C, 3E and 3F). These results are in accordance with the centromere coupling reported in the Arabidopsis *rad51C* mutant [47]. As seen in the frequency distributions, dynamics of centromere pairing in *xrcc3* and *rad51C* is thus similar to WT with, 51%, 50% and 69%, respectively, of the zygo-pachytene nuclei showing 4 or 5 centromeric signals (Figure 3D to 3F). We note, however, that the distribution is a little more spread out in the mutants than in the wild-type (Figure 3D to 3F) and that the proportion of meiocytes of both type 2 and type 3 in *xrcc3* and *rad51C* (47 and 45%, respectively) is higher than in the wild-type (31%). Centromeres thus pair efficiently in *xrcc3* and *rad51C* mutants, in contrast to chromosome arms. Given that the 180 bp DNA repeat region detected by the centromeric FISH probe is found at all Arabidopsis centromeres, the question remains as to whether or not the observed centromeric pairing involves centromeres of homologous chromosomes?

### Pairing of Homologous Centromeres and Pericentromeric Chromosomal Regions in *xrcc3* and *rad51C* Mutants

**Figure 4:**
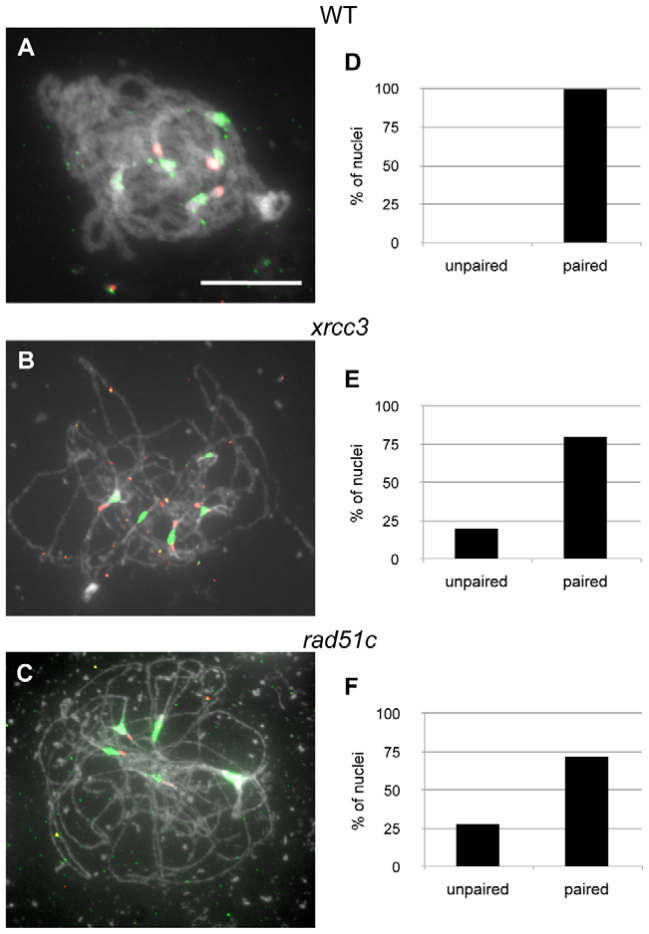
Homologous pairing of centromeres and 5S rDNA loci in *xrcc3 and rad51C* mutants. WT pachytene (A) and *xrcc3* (B) and *rad51C* (C) zygo-pachytene figures showing 5 centromeric signals (green) and three pericentromeric 5S rDNA signals (red). (Scale bar: 10 µm.). (D to F) Percentages of meiocytes with paired 5S rDNA loci in wild-type (D, n = 32), *xrcc3* (E, n = 20), and *rad51C* (F, n = 68).

5S rDNA loci are present near to the centromeres of chromosomes 3, 4 and 5 of the Columbia ecotype of Arabidopsis [52]. As a result, fully synapsed homologous chromosomes in pachytene nuclei show 3 distinct 5S rDNA FISH foci (Figure 4). To quantify the extent of homologous or non-homologous centromere coupling, we examined pachytene meiocytes using FISH with both the 180 bp repeat and 5S rDNA probes. Three 5S rDNA foci thus indicate homologous centromere pairing and more than three, non-homologous centromere coupling. Zygo-pachytene figures in the mutants showing more than 5 or less than 3 centromeric signals were excluded from the analysis as centromere pairing was not yet complete in these meiocytes. Meiocytes exhibiting 2 or 3 5S rDNA signals were considered as homologously paired while meiocytes with more than 3 5S rDNA signals and 4/5 centromere signals were considered as non-homologously coupled.

As shown in Figure 4, 80% of *xrcc3* (n = 20) and 72% of *rad51C* (n = 68) meiocytes showed homologous pairing. Although reduced compared to the wild-type (100% paired), homologous centromere pairing does clearly not fully depend on Xrcc3- and Rad51C-dependent recombination in Arabidopsis.

**Figure 5:**
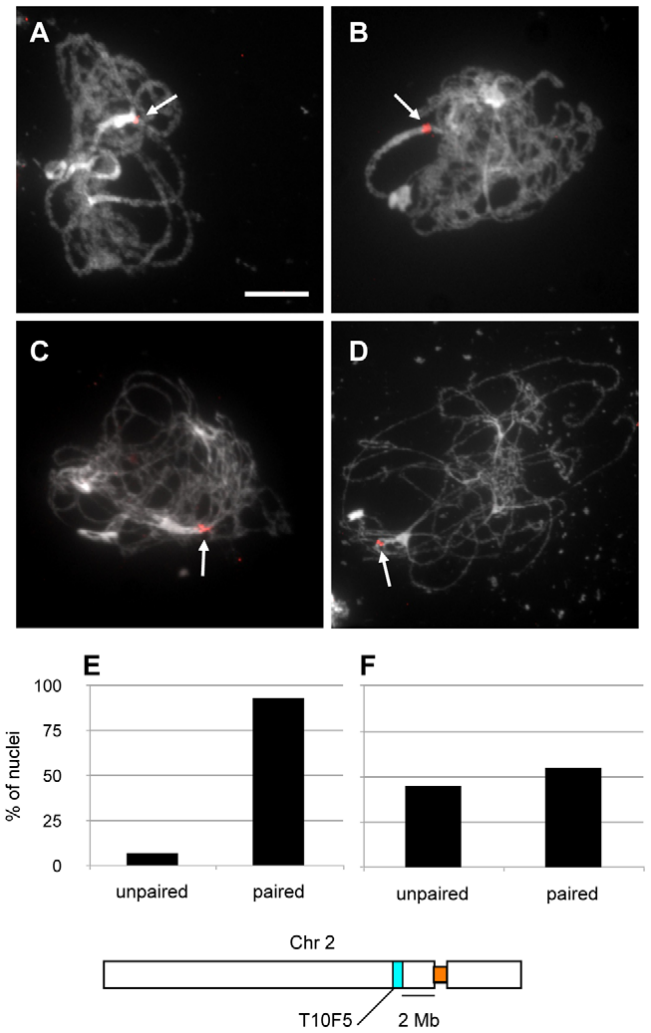
Homologous peri-centromeric pairing of chromosome 2 in *xrcc3* mutants. WT pachytene (A) and *xrcc3* zygo-pachytene (B to D) showing one, or two juxtaposed, chromosome 2 BAC probe T10F5 signals (red). (Scale bar: 10 µm.). Percentages of pairing of chromosome 2 BAC probe T10F5 in pachytene of wild-type (E) (n = 14) and zygo-pachytene of *xrcc3* mutant (F) meiocytes (n = 53).

In order to confirm our observations and to exclude the possibility that homologous pairing is restricted only within these repeated regions, we monitored homologous pairing in *xrcc3* mutants using BAC-FISH with a unique pericentromeric probe (T10F5) located on the long arm of chromosome 2. Two foci were observed in leptotene and early zygotene in both wild-type and *xrcc3* mutants. Beginning at late zygotene and extending through pachytene, pairing proceeds and a single focus is seen in more than 90% of the wild-type meiocytes (Figure 5; Table 2). Homologous pairing was confirmed in *xrcc3* mutants, with 55% of zygo-pachytene stage meiocytes showing a single focus (29 of 53 nuclei; Figure 5 and Table 2). We note that these results underestimate the homologous pairing since, in contrast to 5S rDNA analyses, chromosome 2 foci were monitored in all zygo-pachytene nuclei irrespective of the number of centromere foci. These results thus corroborate the 5S-rDNA FISH (above) showing that rad51 paralogue mutants, *xrcc3* and *rad51C*, show homologous centromere pairing. Strikingly, as seen in Figure 5B, this pairing can extend well into the euchromatic regions flanking the centromeric heterochromatin (the BAC probe used in this analysis lies 2 Mbp from the centromere repeat region).

**Table 2:** Chromosome 2 centromere pairing in meiotic prophase I in WT and *xrcc3* plants.

	probe T10F5			
	Leptotene		Zygo-pachytene	
	unpaired	paired	unpaired	paired
WT	100% (2)	0% (0)	7% (1)	93% (13)
*xrcc3*	91% (31)	9% (3)	45% (24)	55% (29)

Percentages of WT and *xrcc3* meiocytes showing paired or unpaired signals in FISH using a probe targeting chromosome 2 pericentromere. Numbers of nuclei are given in brackets.

### DMC1 Is the Key Recombinase for Efficient Homologous Centromere Pairing

This study was originally undertaken following our observation of meiotic phenotypes of the Arabidopsis *xrcc3* and *rad51C* mutants [44]–[46]. These results have been confirmed and extended by other authors, and strikingly, a similar phenotype is also observed in the *rad51* mutant [32], [50], [53]. This raises the possibility that the homologous centromere pairing we observe in Arabidopsis *xrcc3* and *rad51C* mutant plants is also independent of the Rad51 protein. In order to verify this we carried out experiments to determine which recombination pathway is involved in the homologous centromere pairing.

**Figure 6:**
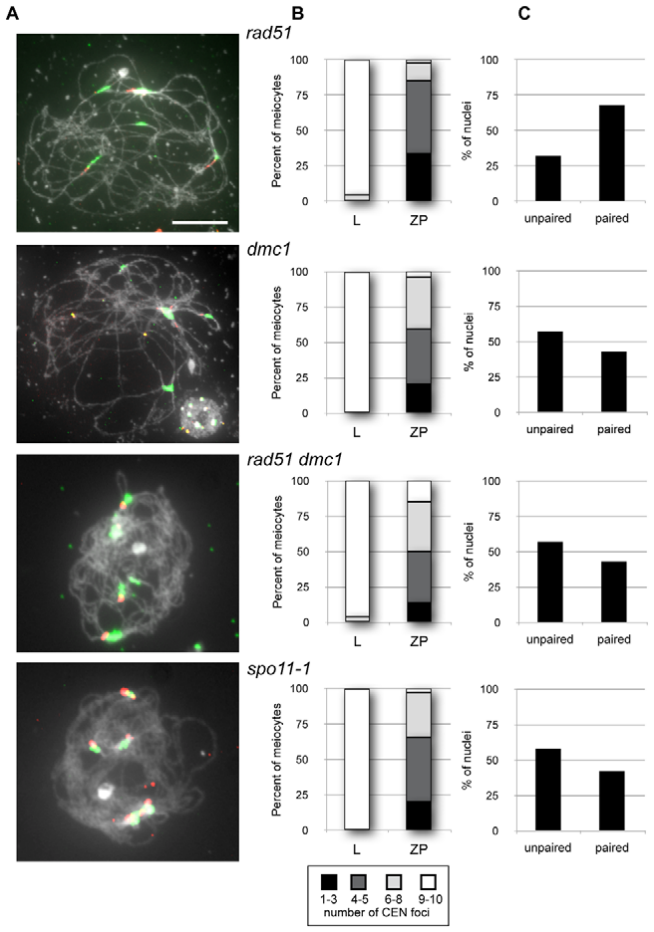
Pairing of centromeres and 5S rDNA loci in *rad51*, *dmc1*, *rad51 dmc1*, and *spo11-1* mutants. (A) Zygo-pachytene of *rad51*, *dmc1*, *rad51 dmc1* and *spo11-1* showing centromeres (green) and three pericentromeric 5S rDNA signals (red) in *rad51* or more in *dmc1*, *rad51 dmc1* and *spo11-1* mutants. (Scale bar: 10 µm.). (B) shows progression in centromere coupling through prophase I for each mutant with 8 to 10 (fully unpaired), 1 to 3 (clustering), 6 to 7 (partially paired) and 4 or 5 centromeres (complete pairing). (C) shows percentages of pairing of 5S rDNA loci in zygo-pachytene nuclei of each mutant. Homologous centromere pairing is observed in *rad51* whereas non-homologous centromere coupling is predominant in *dmc1*, *rad51 dmc1* and *spo11-1* mutants.

Following the approach used for the Rad51 paralogue mutants, we monitored numbers of centromeric and 5S rDNA foci through meiotic prophase I in *rad51*, *dmc1* and *spo11-1* mutants (Figure 6). Complete centromere pairing with 4 or 5 centromeric signals was observed in 51% of the *rad51* zygo-pachytene nuclei, a value similar to that observed for the *xrcc3* and *rad51C* mutants (Figure 3). The *dmc1*, double *rad51 dmc1* and *spo11-1* mutants also show similar levels of centromere pairing, with 39%, 36% and 46%, respectively (Figure 6A and 6B and Table 3). As noted above for the RAD51 paralogue mutants, the frequency distributions of centromeric foci in the mutants are very similar, although slightly more spread out, than in the wild-type (compare Figure S2 with Figure 3D–3F).

**Table 3:** Mean numbers of centromere FISH signals though prophase I in recombination defective lines.

	Interphase/Leptotene	Zygotene	Pachytene
WT	9.3±0.8 (33)	3.9±1.2 (26)	3.9±0.9 (36)
*xrcc3*	9.4±0.6 (21)	4.6±1.5 (45)	-
*rad51C*	9.3±1.0 (101)	5.0±1.5 (172)	-
*rad51*	9.4±0.9 (68)	4.2±1.5 (255)	-
*dmc1*	9.3±0.7 (15)	4.9±1.8 (49)	-
*rad51 dmc1*	9.1±1.0 (51)	5.4±1.8 (94)	-
*spo11-1-2*	9.5±0.7 (38)	5.1±1.4 (70)	-

In all mutants, zygotene and pachytene figures were grouped into the zygo-pachytene stage. Numbers of meiocytes analysed are given in brackets.

These data clearly suggest the existence of a centromere coupling mechanism that is independent of recombination. To test whether or not this concerns centromeres of homologous chromosomes, we monitored the number of 5S rDNA signals in zygo-pachytene mutant meiocytes. These data show the existence of a SPO11-dependent process being responsable for homologous centromere pairing, with *rad51*, *xrcc3* and *rad51C* mutants not being significantly different to the WT (Table 4).

**Table 4:** Mean numbers of 5S rDNA FISH signals through prophase I stages in recombination defective lines.

	Interphase/Leptotene	Zygotene	Pachytene	Significance**
WT	5.4±0.7 (27)	2.9±1.1 (18)	2.6±0.7 (29)	
*xrcc3*	5.4±0.6 (20)	3.0±0.9 (43)	-	ns
*rad51C*	4.9±1.1 (73)	3.2±1.1 (137)	-	ns
*rad51*	5.5±0.7 (13)	3.0±1.0 (197)	-	ns
*dmc1* *	3.8±0.6 (15)	3.0±0.9 (33)	-	*
*rad51 dmc1* *	3.9*±0.7 (30)	2.9*±1.0 (42)	-	*
*spo11-1-2*	5.8±0.5 (32)	3.9±1.0 (65)	-	P<0.0001

In all mutants, zygotene and pachytene figures were grouped into the zygo-pachytene stage. Numbers of meiocytes analysed are given in brackets.

*: The *dmc1* mutant is in the Ws Background and 5S rDNA loci are located only on chromosomes 4 and 5 (see Materials and Methods).

**: Unpaired t-test with WT (ns = not significant, P>0.05).

**Figure 7:**
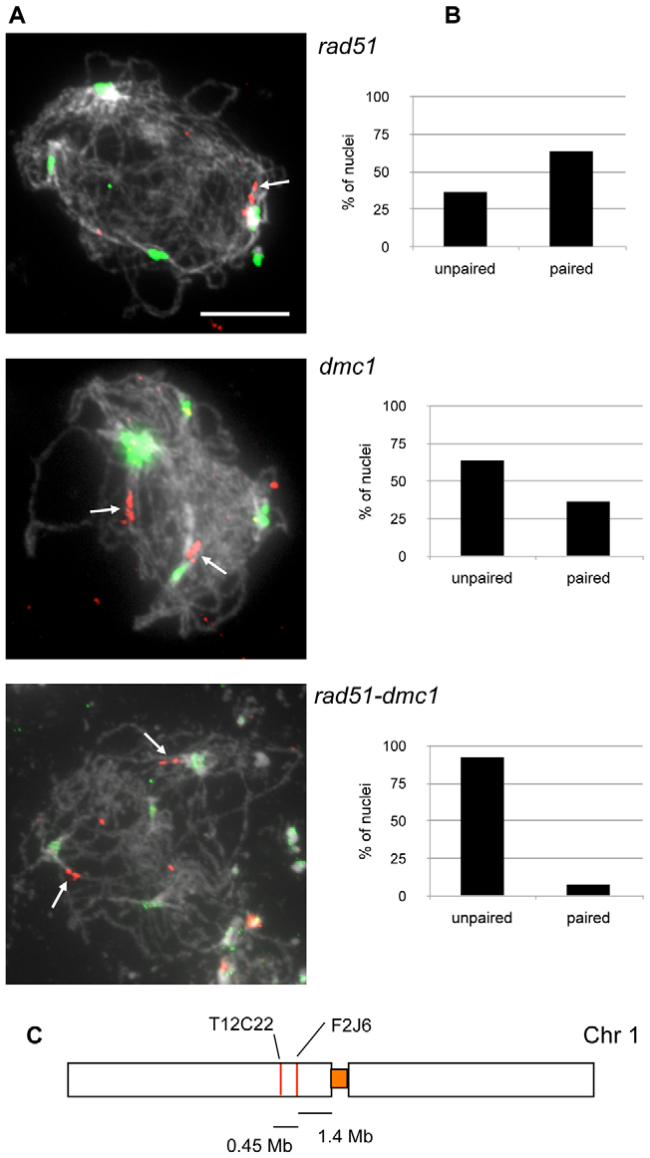
Centromeric pairing of chromosome 1 in *rad51*, *dmc1*, and *rad51 dmc1* mutants. (A) Zygo-pachytene of *rad51*, *dmc1*, and *rad51 dmc1* showing centromeric signals (green) and one or two chromosome 1 BAC signals (red). (Scale bar: 10 µm.). (B) shows percentage of pairing of chromosome 1 BAC probes T10F5 in zygo-pachytene of *rad51* (n = 14), *dmc1* (n = 16), and *rad51 dmc1* (n = 39) mutants. (C) The chromosome BAC probes used in these FISH experiments are depicted below the images.

That the *rad51*, *xrcc3* and *rad51C* mutants do not show the SPO11-dependent effect implies that this homologous centromere pairing principally involves DMC1. We thus dissected the relative roles of DMC1 and RAD51 by monitoring numbers of 5S rDNA foci in nuclei showing 4 to 5 centromeric signals (full centromere coupling, see above). The *rad51* mutant gives values similar to those reported above for the *xrcc3* and *rad51C* mutants, with 68% (n = 101) of zygo-pachytene figures showing homologous centromere pairing (Figure 6C). In contrast, non-homologous centromere coupling was predominant in *dmc1*, *rad51 dmc1* and *spo11-1* mutants, with homologous centromere pairing observed in 43% of *dmc1* (n = 14), 41% of *rad51 dmc1* (n = 17) and 36% of *spo11-1* (n = 36) mutants (Figure 6C). To confirm these data, BAC-FISH analyses were performed in the *rad51*, *dmc1*, and *rad51 dmc1* mutants. Pericentromeric pairing was analysed using BAC-FISH with two adjacent pericentromeric probes, 1.4 to 1.85 Mbp from the centromere of chromosome 1 (Figure 7). As expected, the majority of *rad51* meiocytes with full centromere pairing showed a single BAC-FISH focus and thus homologous centromere pairing (64%, n = 14; Figure 7). In contrast, only 38% of the *dmc1* meiocytes analysed (n = 16) displayed homologous pairing and this dropped to 10% in the *rad51 dmc1* double mutant (n = 39; Figure 7).

Meiotic pairing of homologous centromeres in Arabidopsis thus depends upon Spo11 induced recombination and primarily requires DMC1, but is largely independent of Rad51 and the Rad51 paralogues.

## Discussion

Proper pairing of homologous chromosomes is necessary for proper disjunction at the first meiotic division and thus for the production of functional gametes. This pairing is mediated in the majority of eukaryotes by recombination during the first meiotic prophase and, although the subject of considerable interest, the molecular mechanisms ensuring that chromosomes pair only with their homologues are not fully understood. In the work presented here we have analysed meiotic chromosome pairing in recombination mutants of Arabidopsis and show a differing requirement for these proteins in pairing of pericentromeric regions and chromosomal arms regions during meiotic prophase. We confirm the existence of a non-homologous centromere coupling mechanism in Arabidopsis, independent of the formation of double-strand breaks by SPO11, and RAD51- and DMC1-dependent recombination. Establishment and stabilisation of pairing of homologous centromeric and pericentromeric regions depends principally upon DMC1, while pairing and synapsis of euchromatic chromosome arms of homologues requires the presence of RAD51 and the RAD51 paralogues, XRCC3 and RAD51C.

### Centromere Coupling in Arabidopsis Is Independent of Recombination

Homologous pairing and synapsis is achieved through recognition and proper alignment of homologous chromosomes. With some notable exceptions [54], [55] recombination is clearly required for this, but prior to recombination, different homology-independent interactions have been shown to position and pre-align homologues, thus perhaps preparing the ground for recombination and full homologous synapsis.

The role of telomeres in homologous chromosome positioning and alignment is well established in a number of organisms (reviewed by [7], [56]), although many questions remain concerning the mechanisms involved and the extent to which telomeres participate in pairing is unclear. Of particular relevance here, although Arabidopsis telomeres associate with the nucleolus in meiotic prophase, no telomere “bouquet” is observed [51].

Pairing between non-homologous centromeres, or centromere coupling [11], in early meiotic prophase has been reported in many organisms (reviewed by [16]). In budding yeast, centromere pairing is tightly linked to recombination and initiation of synapsis [9]–[12], [16] and can be sufficient in itself to mediate proper segregation [57]. Non-homologous centromere coupling does not depend upon Spo11-induced recombination, but is dependent on the Zip1 protein. Spo11 and hence initiation of recombination, is however needed for the transition to homologous centromere pairing [10], [11]. Recent results from Drosophila show that centromere clustering initiates SC formation and synapsis of homologous chromosomes in Drosophila female meiosis [58], [59].

In contrast to the pre-meiotic centromere pairing described in onions [60] and wheat [14], Arabidopsis centromeres remain separate until leptotene, when they transiently cluster, separate and initiate pairing during zygotene. Arabidopsis centromere pairing completes during pachytene and is released in diplotene/diakinesis [51]. As summarised in Table 5, meiotic non-homologous centromere coupling has been observed in a number of Arabidopsis mutants affecting meiotic chromosome cohesion, synapsis and recombination, such as the *phs1* mutant [15] (full references are given in Table 5). Only SYN1/REC8, a member of the meiotic cohesin complex, has been shown to be required for centromere coupling in plants, with 10 centromeres being visible throughout prophase I in Arabidopsis *syn1* meiocytes [61]. Similar but less conclusive results are also seen in the mutant of the Arabidopsis SKP1 homologue, ASK1, implicated in a number of early meiotic nuclear reorganisation events [62], [63].

**Table 5:** Arabidopsis mutants for which meiotic centromere behaviour has been reported.

	CEN coupling	5S rDNA pairing	pericentromeric pairing	chromosome arms or telomeric pairing	ASY1 localisation	ZYP1 localisation	TEM of SC	references
WT	+	100%	90%	90–100%	normal	normal	normal	
*xrcc3, rad51C*	+	70%	55%	10–20%	normal	Foci & short stretches	short filaments	This work; [43], [44], [46], [47], [50]
*rad51*	+	70%	64%	-	normal	Foci & short stretches	short filaments	This work; [30], [32], [50]
*dmc1*	+	45%	36%	-	normal	Foci & short stretches	nd	This work; [29], [30], [50]
*rad51 dmc1*	+	45%	10%	-	normal	Foci & short stretches	nd	This work; [30], [50]
*mnd1*	+	nd	nd	-	normal	Foci & short stretches	nd	[50], [78], [79]
*ahp2*	+	nd	nd	-	normal	Foci & short stretches in NOR-bearing arms	short filaments	[80], [81]
*phs1*	+	45%	nd	-	normal	Foci & short stretches (complete synapsis in maize)	full synapsis between non-homologous chromosome in maize	[15], [85]
*rad50*	+	nd	nd	-	nd	nd	nd	[86]
*asy1*	+	nd	nd	paired telomeres	none	numerous foci	nd	[31], [51]
*spo11*	+	45%	nd	nd	normal	No ZYP1	nd	This work; [82]
separase *(aesp, rsw4)*	+	nd	nd	nd	nd	nd	nd	[62], [87]
*swi1*	+	nd	nd	-	Altered axis formation	nd	nd	[88], [89]
*skp1 (ask1)*	−	alteration in rDNA structure	nd	paired telomeres	nd	nd	nd	[62], [63]
*syn1/rec8*	−	nd	nd	-	nd	nd	nd	[61]

“+” indicates that centromere coupling has been observed. When available, pairing data obtained by FISH analyses are listed. 5S rDNA pairing (except for *phs1*) and pericentromeric BAC pairing data are form this work. Data for chromosome arm and telomeric pairing have been obtained from this and cited works. Analyses of Synaptonemal Complex (SC) by Transmission Electron Micrography (TEM) are also listed.

In this work we show that meiotic prophase I coupling of centromeres of non-homologous chromosomes does occur in Arabidopsis *spo11-1*, *rad51*, *dmc1* and *rad51 dmc1* mutants. Notwithstanding the presence of long repetitive DNA homologies at the different Arabidopsis centromeres (see introduction), this process thus does not depend upon the induction of DNA breaks by SPO11, nor the RAD51/DMC1 recombination machinery in Arabidopsis.

### RAD51 and XRCC3 Act Together for Homologous Pairing in Chromosome Arms

Meiosis in Arabidopsis *rad51*, *xrcc3* and *rad51C* mutants appears normal up to the zygotene/pachytene stage, however no fully synapsed pachytene figures are observed and later stages present dramatic chromosomal fragmentation and fusion (This work and [32], [43]–[47], [50]. Some pairing however occurs in these mutants with clustering of centromeres and pairing of telomeres reported in *rad51C* and homologous pairing of 5S rDNA loci in *xrcc3* mutants (this work) [46], [47]. Similarly, telomere pairing has also been reported in *asy1* and *atm* mutants [51], [64]. A recent study of a hypomorph *rad51* Arabidopsis knockdown-mutant provided indications of homologous pairing at metaphase I, with half of the bivalents involving homologous chromosomes and this homologous pairing was abolished in a *rad51 dmc1* double mutant [30]. The severe chromosomal fragmentation and fusion observed post zygotene/pachytene means however that some care must be taken with this conclusion. We present here analyses of the roles of recombination in homologous pairing of specific chromosome regions at the zygo-pachytene stage, prior to chromosome fragmentation/fusion. This approach shows that homologous centromeres pair in Arabidopsis *rad51*, *rad51C* and *xrcc3* mutants. Strikingly, this is not the case for the tested euchromatic chromosome arms. Our results furthermore show that this pairing can extend into the euchromatic pericentromeric regions for at least 2 Mb from the centromere (Figure 5B) The Arabidopsis genome is sequenced and chromosome 2 is estimated at 19,698,400 bp (TAIR10; http://www.arabidopsis.org), which means that the chromosome 2 pairing seen in this figure represents at least 10% of the whole chromosome. To further put this in context, the KEGG database (http://www.genome.jp/kegg-bin/show_organism?org=sce) lists the length of chromosome IV of S. cerevisiae (the largest yeast chromosome) as 1,531,933 bp, considerably less than the 2 Mbp centromere-flanking synapsed region observed here in the Arabidopsis *xrcc3* mutant. RAD51, XRCC3 and RAD51C thus act together to promote pairing and synapsis along chromosome arms.

### DMC1 Is the Key Recombinase for Efficient Pairing of Homologous Centromeres during Meiosis

In most eukaryotes, including plants, meiotic homologous pairing and synapsis are intimately linked to homologous recombination between homologous chromosomes (reviews by [4], [23], [65]). Both the RAD51 and the DMC1 recombinases are necessary for the normal progression of meiotic recombination, with however differing roles, and these differences are believed to be key to the specificities of meiotic and mitotic recombination. RAD51 is involved in both mitotic and meiotic homologous recombination between sister chromatids, while DMC1 acts to promote recombination with the sister chromosome during meiosis, but has no identified role in mitosis. These differing meiotic roles of RAD51 and DMC1 are seen in the phenotypes of Arabidopsis *rad51* and *dmc1* mutants : although no bivalents are detected in the *dmc1* mutant the genome remains intact, while absence of RAD51 leads to genome fragmentation and sterility [29], [32]. Absence of the RAD51 paralogues XRCC3 and RAD51C results in a similar phenotype to *rad51*, with fragmentation of the genome following the zygotene/pachytene stage [43], [44], [46], [47]. The earlier loading of DMC1 compared to RAD51 [31] and the presence of only (intact) univalents in *dmc1* mutants, implies that DSBs are repaired using the sister chromatid as a template and thus that DMC1 functions to favour interhomolog repair [29], [30], [33], [50]. Similarly, recent work on meiosis in a *dmc1* mutant of Tetrahymena has shown the suppression of crossing-over associated with efficient RAD51-dependent repair of DSBs [66]. Studies in yeast led to an asymmetrical strand invasion model in which Dmc1 and Rad51 are loaded on opposite sides of the break [67], [68]. The favoured homology search model is that Dmc1 first invades the homologous partner and that Rad51 acts to stabilise and extend the strand invasion intermediates (see review by [23]).

**Figure 8:**
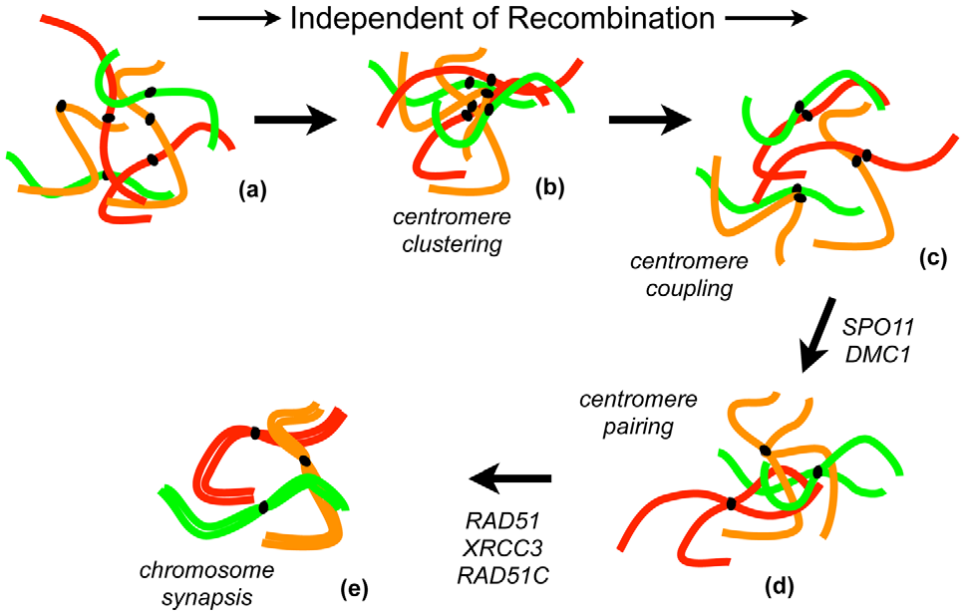
Roles of recombination in chromosome pairing and synapsis during meiotic prophase I in Arabidopsis. In meiotic interphase and leptotene, chromosomes are decondensed and centromeres are dispersed in the nucleus (a). Centromeres cluster in early zygotene (b), separate and couple non-homologously (c), until homologous centromere pairing is established (d), following which arms pair and full synapsis is established. The transition from centromere coupling to homologous centromere pairing requires initiation of recombination by SPO11 and DMC1. In absence of these proteins only coupling of centromeres of non-homologous chromosomes will occur leading to the formation of univalents at metaphase I. Once homologous centromere pairing has been established, pairing and synapsis along chromosome arms will proceed with the help of RAD51 and its paralogues RAD51C and XRCC3 (e). Arabidopsis telomeres have been shown to pair and cluster at the nucleolus during early meiosis (see [64]). However, their behaviour in these recombination mutants is not known and telomere associations have not been included in the diagram for clarity.

As described above, we have confirmed that meiotic coupling of non-homologous centromeres in Arabidopsis occurs independently of recombination. This is not however the case with the transition to homologous centromere pairing, which we show to be dependent on the initiation of recombination by SPO11. Surprisingly, pairing of homologous centromeres seen in nearly 70% of meiocytes of *rad51*, *xrcc3* and *rad51C* mutants, but synapsis does not however extend to chromosome arms. Homologous centromere pairing thus is largely independent of RAD51, XRCC3 and RAD51C. In striking contrast, homologous centromere pairing is significantly reduced in the *dmc1* mutant and this effect is even more pronounced in the *rad51 dmc1* double mutant (and *spo11-1*), confirming the primary role of DMC1 and showing a minor role for RAD51. Such a role for RAD51 in supporting DMC1 in Arabidopsis is in accordance with the recent study of the hypomorph *rad51-2* allele [30]. Meiotic pairing of homologous centromeres in Arabidopsis is thus dependent on the initiation of recombination by SPO11 and principally promoted by DMC1. Extension and completion of synapsis to chromosome arms is however dependent upon RAD51 (and XRCC3 and RAD51C). Based on these data, we suggest a model of meiotic chromosome pairing in Arabidopsis in which, following recombination-independent non-homologous centromere coupling, homologous chromosome pairing is initiated at centromeres via a DMC1-dependent interhomolog search mechanism and that pairing is further stabilised and extended along chromosome arms through RAD51-dependent homologous recombination (Figure 8).

### A Role for Heterochromatin in Meiotic Chromosome Pairing?

The observations of differing requirements for RAD51- and DMC1-dependent recombination at heterochromatic centromeres and euchromatic chromosome regions raises the question of whether these differences are centromere-specific or whether they reflect differing behaviour of heterochromatin and euchromatic regions in chromosomal synapsis.

The role for heterochromatin in chromosome pairing during Drosophila female achiasmate disjunction has long been established [69], [70] and studies in maize and *C. elegans* have shown that chromosome pairing is associated with a change in chromatin conformation [71], [72]. In plants, strong evidence for a role of chromatin remodeling in pairing comes from analyses in wheat, where the recognition and pairing of homologous chromosomes at telomeres and centromeres triggers a conformational change in adjacent chromatin that is suggested to further facilitate homologous chromosome recognition and recombination [73]–[75]. Whether such mechanisms also occur in Arabidopsis is unknown. The existence of a centromeric coupling mechanism that is independent of recombination suggests a role for heterochromatin and chromatin structure in the homology search also in Arabidopsis and this may not be restricted to centromeres. In this respect it is interesting to note that in most Arabidopdis ecotypes, cytological observations show the two NORs to be very frequently associated as one large knob [52], [76], [77]. This characteristic association of the nucleolar heterochromatin present on chromosomes 2 and 4 was also observed in all of the mutants studied in this work, indicating that this association is independent of recombination.

Moreover, notwithstanding the non-homologous chromosome pairing observed in Arabidopsis *mnd1* and *hop2/ahp2* mutants [78]–[81], in *ahp2* mutants the NOR-bearing arms of chromosomes 2 and 4 exhibit stabilised homologous pairing and synapsis [80]. These observations strongly suggest a role for heterochromatin in pairing in Arabidopsis. Uncovering the precise role of heterochromatin in meiotic pairing will require further studies such as analysis of mutants with altered heterochromatin configuration or chromatin remodeling capacity.

## Materials and Methods

### Plant Material and Growth Conditions

All *Arabidopsis thaliana* plants used in this study were of Columbia ecotype with the exception of *dmc1* (Wassilewskija). Arabidopsis *xrcc3*, *rad51C*, *rad51*, *dmc1*, *rad51 dmc1*, and *spo11-1-2* mutants and PCR genotyping have been described previously [29], [32], [44], [46], [47], [50], [82]. The *dmc1* allele used in this experiment (*dmc1* and *rad51 dmc1* mutants) comes from the Wassilewskija (Ws) background which only has 2 5S rDNA loci, on chromosomes 4 and 5. The *DMC1* locus is situated on the same chromosome (III) as the 5S rDNA locus and we verified that the *rad51 dmc1* mutant lines used here have 4 rDNA FISH foci in interphase and leptotene nuclei (unpaired chromosomes). Seeds were sown onto soil, stratified in water at 4°C for 2 days and grown in a greenhouse with a 16/8 h light∶dark photoperiod; temperature 23°C; and approximately 60% relative humidity.

### Fluorescent In Situ Hybridisation

#### Chromosome preparation by spreading

Chromosome spreads were prepared according to Ross et al. [77] with the modifications introduced by Fransz et al. [52]. Briefly, whole inflorescences were fixed in absolute ethanol/glacial acetic acid (3∶1) for 3×30 min and stored at 4°C. Immature flower buds were rinsed twice at room temperature in distilled water for 5 min followed by two washes in 1× citrate buffer for 5 min. Buds of appropriate size were selected under a binocular microscope and incubated for 3 hr. 30 mins. on a slide in 100 µl of enzyme mixture (0.3% w/v cellulase (Sigma), 0.3% w/v pectolyase (Sigma) and 0.3% cytohelicase (Sigma) in a moist chamber at 37°C. Each bud was then softened in 15 µl 60% acetic acid on a microscopic slide at 45°C, fixed with ice-cold ethanol/glacial acetic acid (3∶1) and air dried. Slides were then rinsed in Coplin jar for 2 mins. in distilled water, 10 mins. in 4% paraformaldehyde in 1× PBS and finally rinsed for 5 min in distilled water; air-dried and stored at 4°C for further use.

#### Probe preparation and labelling

The following DNA probes were used: a centromeric specific probe (containing two copies of the 180 bp repeat) [83], a 5S rDNA probe, BAC probes T10F5 (chromosome 2 pericentromere), F12C20 (chromosome 2 long arm), F12K11 (chromosome 1 left-arm), F2J6 (chromosome 1 pericentromere), and T12C22 (chromosome 1 pericentromere). BACs were obtained from the Ohio Arabidopsis Stock Center, ABRC (http://abrc.osu.edu).

DNA from BAC clones was prepared using QIAGEN Large-Construct Kit according to the manufacturer's instructions. BAC probes were labelled with either biotin-dUTP or digoxygenin-dUTP using a Nick-Translation kit (ROCHE) following manufacturer's recommendations. The 180 pb and 5S rDNA probes were labelled by PCR incorporation of dUTP-biotin or dUTP-Digoxygenin according to Lysak et al. (2006). Probes were made with 1 µl of PCR product and/or 3 µl of Nick-Translation product, HB50 buffer to 10 µl and 10 µl Dextran Sulfate 20% in HB50. Probes were used either immediately or stored at −20°C until use. In Situ Hybridisation and fluorescence detection of hybridised probes were performed according to Lysak et al. [84].

### Microscopy

All observations were made with a motorised Zeiss AxioImager.Z1 epifluorescence microscope (Carl Zeiss AG, Germany) using a PL Apochromat 100X/1.40 oil objective. Photographs were taken with an AxioCam Mrm camera (Zeiss) and appropriate Zeiss filter sets adapted for the fluorochromes used : filter set 25HE (DAPI), filter set 38HE (Alexa 488) and filter set 43HE (Texas-Red). Captured images were further processed with AxioVision 4.6.2. and Adobe Photoshop CS4 software.

## Supporting Information

Figure S1 ZYP1 localisation is disturbed in *xrcc3* mutants. Male meiocytes stained with DAPI (blue) and the AtZYP1 antibody (green). AtZYP1 extends along the entire length of the chromosome axes in wild-type pachytene. Numerous foci and short stretches of AtZYP1 (arrows) staining are present in *xrcc3* mutants (Scale bar = 5 µm.).
(TIF)

Figure S2 Frequency distributions of centromeric FISH signals in prophase I of *rad51*, dmc1, *rad51 dmc1*, and *spo11-1-2* mutants.
(TIF)
