# Supplementary figures and images for: Differing Requirements for RAD51 and DMC1 in Meiotic Pairing of Centromeres and Chromosome Arms in Arabidopsis thaliana
Source: PLoS Genet. 2012 Apr 19;8(4):e1002636. doi: 10.1371/journal.pgen.1002636 (PMC3330102; doi:10.1371/journal.pgen.1002636)

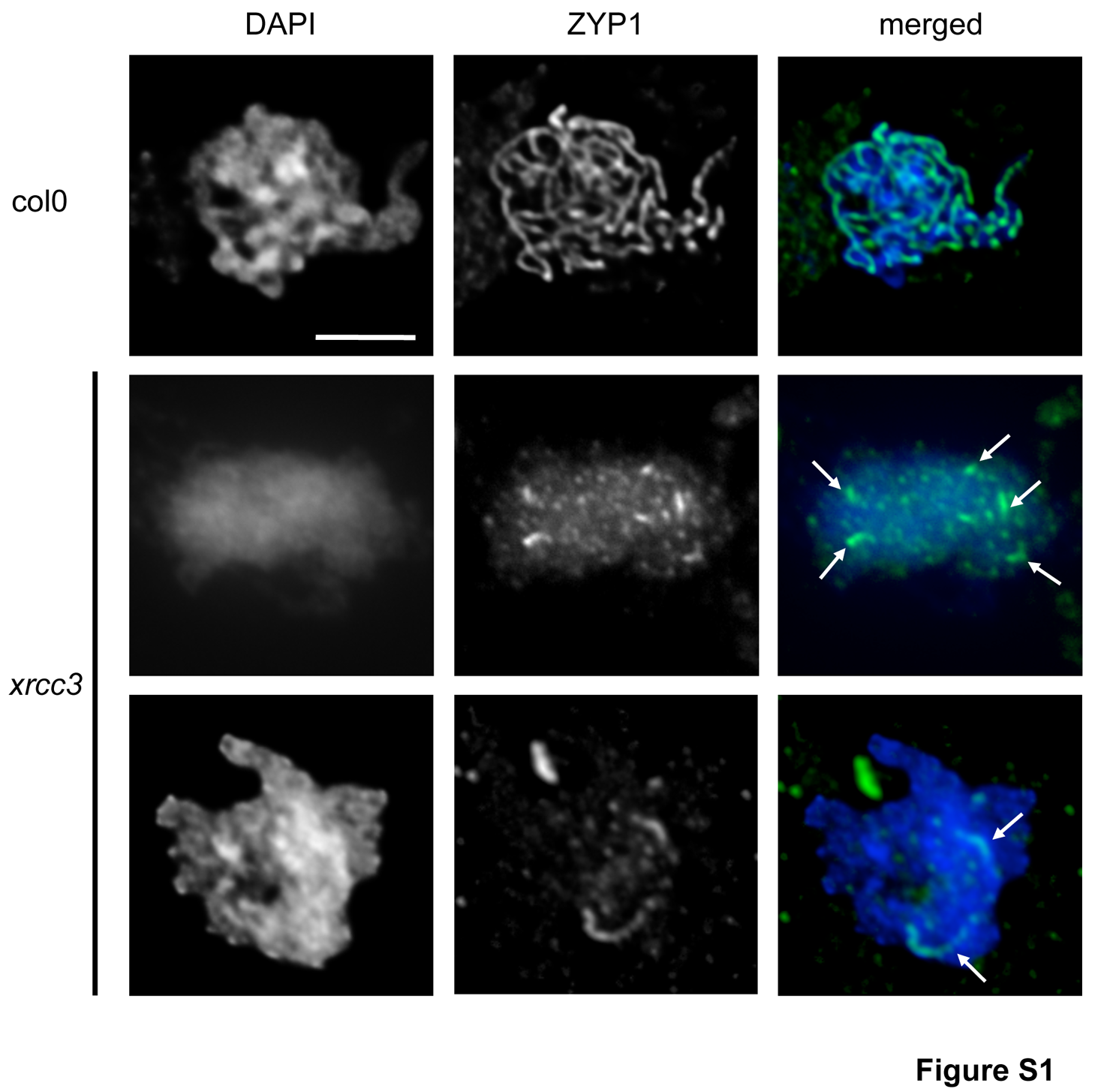

Supplement: Figure S1 — ZYP1 localisation is disturbed in xrcc3 mutants. Male meiocytes stained with DAPI (blue) and the AtZYP1 antibody (green). AtZYP1 extends along the entire length of the chromosome axes in wild-type pachytene. Numerous foci and short stretches of AtZYP1 (arrows) staining are present in xrcc3 mutants (Scale bar = 5 µm.). (TIF) [file pgen.1002636.s001.tif]

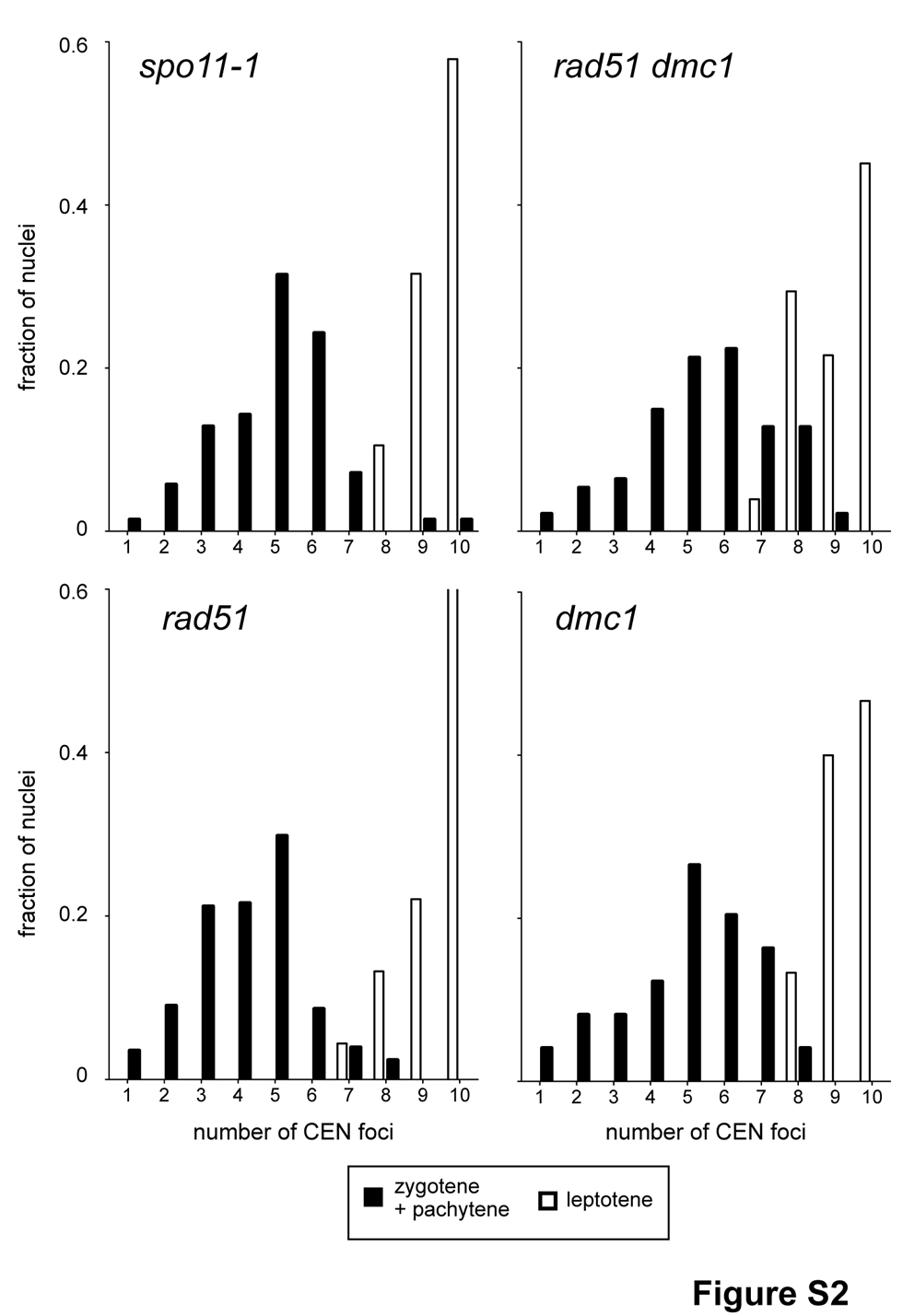

Supplement: Figure S2 — Frequency distributions of centromeric FISH signals in prophase I of rad51, dmc1, rad51 dmc1, and spo11-1-2 mutants. (TIF) [file pgen.1002636.s002.tif]
